# Intervention randomized controlled trials involving wrist and shoulder arthroscopy: a systematic review

**DOI:** 10.1186/1471-2474-15-252

**Published:** 2014-07-25

**Authors:** Kamelia Tadjerbashi, Roberto S Rosales, Isam Atroshi

**Affiliations:** 1 Department of Orthopedics, Hässleholm Hospital, Hässleholm SE-28125, Sweden; 2 Unit for Hand & Microsurgery, GECOT, La Laguna, Tenerife, Spain; 3 Department of Clinical Sciences, Lund University, Lund, Sweden

**Keywords:** Arthroscopy, Wrist, Shoulder, Randomized trials, Jadad scale, Intervention RCT, Systematic review

## Abstract

**Background:**

Although arthroscopy of upper extremity joints was initially a diagnostic tool, it is increasingly used for therapeutic interventions. Randomized controlled trials (RCTs) are considered the gold standard for assessing treatment efficacy. We aimed to review the literature for intervention RCTs involving wrist and shoulder arthroscopy.

**Methods:**

We performed a systematic review for RCTs in which at least one arm was an intervention performed through wrist arthroscopy or shoulder arthroscopy. PubMed and Cochrane Library databases were searched up to December 2012. Two researchers reviewed each article and recorded the condition treated, randomization method, number of randomized participants, time of randomization, outcomes measures, blinding, and description of dropouts and withdrawals. We used the modified Jadad scale that considers the randomization method, blinding, and dropouts/withdrawals; score 0 (lowest quality) to 5 (highest quality). The scores for the wrist and shoulder RCTs were compared with the Mann–Whitney test.

**Results:**

The first references to both wrist and shoulder arthroscopy appeared in the late 1970s. The search found 4 wrist arthroscopy intervention RCTs (Kienböck’s disease, dorsal wrist ganglia, volar wrist ganglia, and distal radius fracture; first 3 compared arthroscopic with open surgery). The median number of participants was 45. The search found 50 shoulder arthroscopy intervention RCTs (rotator cuff tears 22, instability 14, impingement 9, and other conditions 5). Of these, 31 compared different arthroscopic treatments, 12 compared arthroscopic with open treatment, and 7 compared arthroscopic with nonoperative treatment. The median number of participants was 60. The median modified Jadad score for the wrist RCTs was 0.5 (range 0–1) and for the shoulder RCTs 3.0 (range 0–5) (p = 0.012).

**Conclusion:**

Despite the increasing use of wrist arthroscopy in the treatment of various wrist disorders the efficacy of arthroscopically performed wrist interventions has been studied in only 4 randomized studies compared to 50 randomized studies of significantly higher quality assessing interventions performed through shoulder arthroscopy.

## Background

Although arthroscopy of upper extremity joints was initially introduced mainly for diagnostic purposes it is being increasingly used for therapeutic interventions [1]. For example, wrist interventions performed through arthroscopy include, among others, excision of wrist ganglia, treatment of acute fractures and of non-unions, ligament repair and reconstructions, repair or debridement of the triangular fibrocartilage complex, ulnar head resection, partial or total removal of carpal bones, and joint fusions [1,2]. A recent study on musculoskeletal upper extremity ambulatory surgery in the United States estimated that 272,148 rotator cuff repairs, 257,541 shoulder arthroscopies excluding those for cuff repairs, 3686 elbow arthroscopies, and 25,250 wrist arthroscopies were performed in 2006 [3]. Arthroscopic interventions generally require special equipment and substantial surgical training and may thus be associated with higher costs than open procedures [4]. In addition, arthroscopic procedures may be associated with various complications [5]. Arthroscopic interventions may, however, be more cost-effective if their efficacy is superior to that of non-arthroscopic treatments or if they have similar efficacy but provide additional benefit, such as quicker recovery or lower morbidity. There is strong agreement that good-quality randomized controlled trials (RCTs) are the gold standard for assessing treatment efficacy and that they provide higher level of evidence than observational studies [6]. We reviewed the literature for intervention RCTs involving wrist arthroscopy, and for comparison, shoulder arthroscopy, hypothesizing that the quality of wrist and shoulder RCTs are similar.

## Methods

We performed a systematic review of the literature for randomized or quasi-randomized clinical trials in which at least one arm was an intervention performed through wrist arthroscopy or shoulder arthroscopy. An experienced researcher searched for articles published up to December 2012 in the databases PubMed and Cochrane Library. The search was conducted in accordance with the Preferred Reporting Items for Systematic Reviews and Meta-Analyses (PRISMA) guidelines [7]. The search strategy was applied to PubMed and optimized for the Cochrane database (Additional file 1). We included all RCTs written in English, Spanish, or German. We omitted conference abstracts. We checked the references of the initially included articles to identify other potentially relevant studies and subjected them to a similar selection process.

Three researchers reviewed the selected articles (each article reviewed by at least two researchers) and recorded the following data: the country where the study was conducted, the condition for which the interventions were done, the randomization method, the number of randomized participants, the time of randomization, the outcomes measures used, blinding, and description of dropouts and withdrawals. When appropriate we grouped the conditions for which the interventions were done into diagnostic categories. As a measure of RCT quality we used the Jadad scale [8] as modified by Gummesson et al. [9]. The scale considers the randomization method, blinding and description of dropouts/withdrawals, yielding a score from 0 (lowest quality) to 5 (highest) [9]. A study that describes an appropriate randomization method (such as computer-generated sequence or a random-number table) is awarded 2 points while a study that does not report the randomization method or reports an inappropriate method (such as order of presentation or medical record number) is not awarded any points. Similarly a study that reports blinding (single or double) using an appropriate method is awarded 2 points while use of an inappropriate blinding method or absence of blinding does not yield any points. The blinding method was considered appropriate if the article specified whom the blinding involved and, depending on the nature of the interventions, possible additional measures to ensure the blinding (for example, stating that blinding involved an assessor and that the surgical area was covered during patient assessment or that identical incisions were used for the different surgical procedures). Description of any dropouts or withdrawals (or a statement that no dropouts/withdrawals occurred) is awarded 1 point. The grading according to the modified Jadad scale was done by two researchers independently and any disagreements were resolved by discussion until consensus was reached.

The median modified Jadad scores were calculated for the wrist and shoulder RCTs and were then compared with the Mann–Whitney test. A p-value of less than 0.05 was considered to indicate statistical significance.

## Results and discussion

### Results

The Medline search showed that the first publications in which wrist arthroscopy or shoulder arthroscopy were mentioned appeared in the late 1970s.

### Wrist arthroscopy

**Figure 1:**
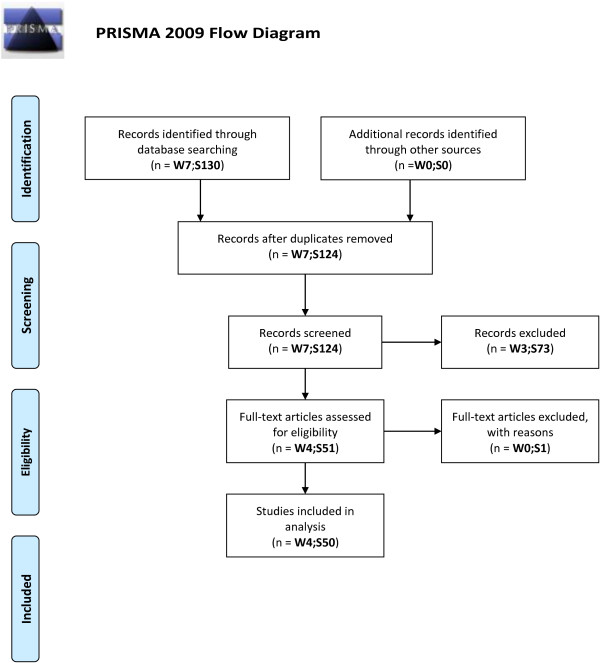
**RCTs involving wrist arthroscopy or shoulder arthroscopy – inclusion and exclusion flow diagram.** Details of the inclusion and exclusion process of the finally selected intervention randomized controlled trials in which at least one arm involved wrist arthroscopy or shoulder arthroscopy; shown in a PRISMA flow diagram. W = number of wrist arthroscopy articles; S = number of shoulder arthroscopy articles.

Of 7 possible RCTs obtained in the search, 3 were excluded because they involved postoperative analgesia, leaving 4 intervention RCTs eligible for inclusion (Figure 1; Additional file 2). The 4 RCTs (Table 1) involved Kienböck’s disease (arthroscopic versus open surgery), dorsal wrist ganglia (arthroscopic versus open excision), volar wrist ganglia (arthroscopic versus open excision), and distal radius fracture (arthroscopically- and fluoroscopically-assisted versus fluoroscopically-assisted reduction, followed by fixation). The number of participants in the 4 studies was 16, 50, 72, and 40, respectively (median 45).

**Table 1:** Details of the intervention randomized controlled trials in which at least one arm involved wrist arthroscopy or shoulder arthroscopy

Author* (first) yr	**Country**	**Diagnosis**	**Intervention 1**	**N 1**	**D/W**	**Intervention 2**	**N 2**	**W/D**	**Randomization method**	**Time of randomization**	**Outcomes**	**Blinding**
**Wrist**												
Kang 2008	USA	Dorsal ganglion	Arthroscopic excision	41	13	Open excision	31	8	Medical record Identifier (odd/even)	At presentation	Recurrence, residual pain, complications	NR
Leblebicioglu 2003	Turkey	Kienböck’s disease	Open scaphocapitate fusion and lunate revascularization	8	NR	Arthroscopic scapho-capitate fusion and capitate pole excision	8	NR	Last digit of Medical record (odd/even)	NR	Operative time, LOHS, time to fusion, ROM, grip, RTW	NR
Rocchi 2008	Italy	Volar ganglion	Open excision	25	2	Arthroscopic excision	25	1	Sealed envelopes	NR	ROM, grip, scar, pain, residual symptoms, recurrence	NR
Varitimidis 2008	Greece	Intra-articular distal radius fracture	Arthroscopic and fluroscopic assisted reduction + external fixation and percutaneous pinning	20	NR	Fluroscopic assisted reduction + external fixation and percutaneous pinning	20	NR	Sealed envelopes	NR	Mayo wrist score, DASH (primary), clinical wrist instability, grip, ROM, radiographs	NR
**Shoulder**												
Archetti Netto 2012	Brazil	Traumatic anterior instability + isolated Bankart lesion	Arthroscopic repair	22	5	Open repair	28	3	Computer; Sealed envelopes	At surgery	DASH (primary), UCLA, Rowe, ROM	NR
Barber 2012	USA, Canada	Large rotator cuff tear	Arthroscopic single- row repair + acellular human dermal matrix augmentation	22	NR	Arthroscopic single row repair	20	NR	Sealed envelopes	At surgery	ASES, UCLA, Constant, MRI, ROM, strength	Assessor (radiologist)
Berth 2010	Germany	Massive rotator cuff tear	Arthroscopic partial rotator cuff repair	21	NR	Arthroscopic debride-ment + subacromial decompression	21	NR	Patient's option	NR	Constant, ROM, pain, DASH, ultrasound	NR
Bottoni 2002	USA	Acute, traumatic, first-time shoulder dislocations in young athletes	Arthroscopic stabilization	10	1	Nonoperative treatment (4 wks immobilization followed by supervised rehabilitation program)	14	2	Last digit social security number (odd/even)	NR	Recurrent instability, SANE, L'Insalata shoulder evaluation, satisfaction	NR
Bottoni 2006	USA	Recurrent anterior shoulder instability	Arthroscopic stabilization	32	0	Open stabilization	32	3	Sealed envelopes	NR	ROM, stability, SANE, SST, WOSI, UCLA, Rowe	Assessor (physiotherapist)
Brox 1993	Norway	Impingement syndrome (stage II)	Arthroscopic acromioplasty	45	13	Supervised exercises; Placebo laser	50;30	8;4	NR	Mean 2 months before treatment	Neer shoulder score (primary), pain	Assessor
Burks 2009	Australia	Full-thickness rotator cuff tear	Arthroscopic single-row rotator cuff repair	20	0	Arthroscopic double-row rotator cuff repair	20	0	Random number Generator; Sealed envelopes	At surgery	UCLA, MRI, Constant-Murley, WORC, SANE, ASES, ROM, strength	Assessors (radiologist and examiner)
Charron 2007	USA	Distal clavicle osteolysis or post-traumatic acromio- clavicular arthrosis without instability	Arthroscopic distal clavicle resection with a direct approach	19	1	Arthroscopic distal clavicle resection with an indirect subacromial approach	19	3	Order of enrollment (odd/even)	At enrollment	ASES, ATH, time to full return to sports	NR
Chen 2010	China	Frozen shoulder	Arthroscopic release of anterior capsular structures	42	1	Arthroscopic release extended inferiorly and posteriorly	32	3	Computer; Sealed envelopes	At surgery	Constant, ROM	Patients and Assessors
De Carli 2012	Italy	Idiopathic adhesive shoulder capsulitis	Arthroscopy arthrolysis and shoulder manipulation	25	2	Glenohumeral steroid injections	21	0	NR	NR	ROM, ASES, UCLA, SST, Constant-Murley	NR
Dezaly 2011	France	Rotator cuff tear in the over-60s	Arthroscopic biceps acromioplasty-tenotomy and repair	71	3?^†^	Arthroscopic biceps acromioplasty-tenotomy	71	12?^†^	NR	Day before surgery	Constant, ultrasound tendon healing	NR
Elmlund 2009	Sweden	Recurrent shoulder instability	Arthroscopic reconstruction with polygluconate-B polymer	20	4	Arthroscopic reconstruction with poly-L-lactic acid polymer tack implants	20	3	Sealed envelopes	Just before surgery	Radiographs, CRP, Constant, Rowe, apprehension test, strength, ROM, recurrence of instability	Assessor (radiologist)
Fabbriciani 2004	Italy	Traumatic anterior shoulder instability	Arthroscopic repair	30	NR	Open repair	30	NR	Computer	At surgery	Constant, Rowe	NR
Franceschi 2008	Italy	Rotator cuff tear and a type II SLAP lesion in the over-50s	Arthroscopic repair of both lesions	31	2	Arthroscopic rotator cuff tear repair without repair of the SLAP II lesion but with tenotomy of the long head of the biceps	32	5	Random number table; Sealed envelopes	At surgery	UCLA, ROM	NR
Freedman 2007	USA	Refractory acromioclavicular joint pain	Open distal clavicle excision	9	1	Arthroscopic distal clavicle excision	8	1	NR	NR	Pain VAS (primary). modified ASES, SF-36	NR
Gartsman 2004	USA	Full-thickness rotator cuff tear + type 2 acromion	Arthroscopic rotator cuff repair + subacromial decompression	47	NR	Arthroscopic rotator cuff repair without subacromial decompression	46	NR	Random number table	At surgery	ASES	Patients
Grasso 2009	Italy	Full-thickness rotator cuff tear	Arthroscopic single-row rotator cuff repair	40	3	Arthroscopic double-row rotator cuff repair	40	5	Computer	At surgery	DASH, Constant, strength	NR
Gumina 2012	Italy	Large full-thickness posterosuperior rotator cuff tear	Arthroscopic repair with platelet-leukocyte membrane	40	1	Arthroscopic repair	40	3	Randomization list; Sealed envelopes	3 days before surgery	Constant, MRI (primary), SST	Assessors
Haahr 2005	Denmark	Subacromial impingement	Arthroscopic subacromial decompression	45	4	Physiotherapy	45	2	Computer; Sealed envelopes	NR	Constant, pain VAS, ROM, strength, ADL	NR
Henkus 2009	Nether-lands	Primary subacromial impingement without rotator cuff rupture	Arthroscopic subacromial bursectomy	27	1	Debridement of subacromial bursa + arthroscopic acromioplasty	30	0	Automatically generated randomization code	NR	Constant, SST , pain VAS, functional impairment VAS	Assessor and group 1 patients
Hiemstra 2008	Canada	Shoulder instability	Open stabilization	24	0	Arthroscopic stabilization	24	0	Computer	NR	Strength (primary), ASES, ROM	Assessor
Husby 2003	Norway	Impingement syndrome (Neer grade II)	Arthroscopic subacromial decompression	20	5	Open subacromial decompression	19	0	Sealed envelopes	At surgery	UCLA, pain VAS, satisfaction VAS, strength, ROM	Assessor
Kasten 2011	Germany	Supraspinatus tendon rupture	Arthroscopic repair	17	3	Mini-open technique	17	1	Order of enrollment(first 17/ next 17)	NR	NSAID use, pain, Constant-Murley, ASES, MRI	Assessor (radiologist)
Ketola 2009	Finland	Shoulder impingement syndrome	Supervised exercise	70	4	Arthroscopic acromioplasty + supervised exercise	70	2	Computer;Sealed envelopes	NR	Pain VAS, ROM, strength (primary), cost-effectiveness	Assessor (physiotherapist)
Kim 2011	Korea	Rotator cuff tear + asymptomatic acromioclavicular arthritis	Arthroscopic distal clavicle resection with rotator cuff repair	31	2	Arthroscopic rotator cuff repair	52	4	Random number table	NR	ASES, UCLA, pain, AC joint tenderness, cross body adduction test	NR
Kirkley 1999	Canada	First traumatic anterior dislocation	Immediate arthroscopic stabilization	19	0	Immobilization and rehabilitation	21	2	NR	NR	WOSI, ROM, redislocation	Assessor
Koh 2011	South Korea	Rotator cuff tear	Arthroscopic single-row repair	37	6	Arthroscopic double-row repair	34	3	Computer	At surgery	Pain VAS, Constant, ASES, UCLA, re-tear, MRI	Assessors (radiologist and examiner)
Lindh 1993	Sweden	Shoulder impingement	Arthroscopic subacromial decompression	10	0	Open acromioplasty	10	0	NR	NR	Osteophyte recurrence, ROM, UCLA	NR
Ma 2012	Taiwan	Full-thickness rotator cuff tear	Arthroscopic single-row repair	32	5	Arthroscopic double-row repair	32	6	Computer; Sealed envelopes	At surgery	UCLA, ASES, strength, magnetic resonance arthrography	NR
MacDonald 2011	Canada	Full-thickness rotator cuff tear	Arthroscopic repair + acromioplasty	41	9	Arthroscopic repair	45	9	Computer; Sealed envelopes	At surgery	WORC (primary), ASES, revision	Patients and Assessor
Magnusson 2006	Sweden	Post-traumatic shoulder instability	Arthroscopic Bankart reconstruction with polygluconate co-polymer	20	0	Arthroscopic Bankart reconstruction with self-reinforced poly-L-lactic acid polymer	20	0	Sealed envelopes	Just before surgery	Strength, ROM, Rowe, Constant, stability, radiography	Assessor (radiologist)
Milano 2007	Italy	Full-thickness rotator cuff tear	Arthroscopic repair + subacromial decompression	40	3	Arthroscopic repair	40	6	Computer	At surgery	Constant, DASH	NR
Milano 2010	Italy	Recurrent traumatic anterior shoulder instability	Arthroscopic repair with metal suture anchor	39	3	Arthroscopic repair with biodegradable suture anchor	39	5	Random sequence generator; Sealed envelopes	At surgery	DASH (primary),,Rowe, Constant, recurrence	Assessor
Milano 2010	Italy	Full-thickness rotator cuff tear	Arthroscopic repair with metal suture anchor	55	3	Arthroscopic repair with biodegradable suture anchor	55	6	Random sequence generator	At surgery	DASH, Constant	Assessor
Mohtadi 2008	Canada	Full-thickness rotator cuff tear	Open repair	37	8	Arthroscopic acromioplasty with mini-open repair	36	5	Computer; Sealed envelopes	NR	RC-QOL (primary), ASES, SRQ, FSET, ROM, strength	Assessor
Monteiro 2008	Brazil	Traumatic anterior shoulder instability	Arthroscopic repair with anchors loaded with absorbable sutures	25	4	Arthroscopic repair with anchors loaded with nonabsorbable sutures	25	1	Sealed envelopes	NR	Rowe, ASOSS	Assessor
Oh 2011	South Korea	Partial- or full- thickness rotator cuff tear	Arthroscopic repair + HA/carboxymethylated cellulose injection	40	NR	Arthroscopic repair	40	NR	Computer	NR	Pain VAS, PROM, ASES, ultrasonography, CTA	Injection and Assessor
Randelli 2011	Italy	Complete rotator cuff tear	Arthroscopic repair + autologous platelet rich plasma	26	4	Arthroscopic repair	27	4	Computer; Sealed envelopes	At surgery	Pain VAS, SST, UCLA, Constant, strength, MRI	Assessors (radiologist and examiner)
Robinson 2008	UK	First-time traumatic anterior dislocation	Arthroscopic examination and lavage	45	3	Arthroscopic examination and Bankart lesion repair	43	1	Computer; weighted minimization	NR	Recurrence, functional scores, DASH, patient satisfaction, SF-36, WOSI, ROM, cost	Patients and Assessor (physiotherapist)
Rodeo 2012	USA	Full-thickness rotator cuff tear	Arthroscopic repair + platelet-rich fibrin matrix	40	5	Arthroscopic repair	39	7	Sealed envelopes	At surgery	Healing on ultrasound (primary), ASES, L'Insalata, manual muscle testing	Patients and Assessor
Sachs 1994	USA	Impingement syndrome (stage II)	Arthroscopic acromioplasty	22	3	Open acromioplasty	22	0	NR	NR	Pain, function, ROM, strength, RTA, LOHS	NR
Shin 2012	South Korea	Small-medium rotator cuff tear	Arthroscopic repair + acromioplasty	75	15	Arthroscopic repair	75	15	NR	Before surgery	VAS, UCLA, ASES, Constant, MRI, ROM	NR
Shin 2012	South Korea	Partial-thickness articular-sided rotator cuff tear	Arthroscopic repair with transtendon technique	24	0	Arthroscopic repair with full-thickness conversion	24	0	Computer	At surgery	Pain and satisfaction VAS, ASES, Constant, MRI, ROM	Assessors (radiologist and examiner)
Silberberg 2011	Spain	Isolated type II SLAP lesion	Arthroscopic repair with vertical suture	15	0	Arthroscopic repair with horizontal suture	17	0	Minimization	At surgery	Pain and instability VAS, ASES, ROM	Assessor
Spangehl 2002	Canada	Impingement syndrome	Arthroscopic acromioplasty	32	?/25^†^	Open acromioplasty	30	?/25^†^	NR	NR	Pain and function VAS (primary), UCLA, satisfaction, strength	Assessor
Sperber 2001	Sweden	Traumatic anterior shoulder instability	Arthroscopic stabilization	30	NR	Open stabilization	26	NR	Sealed envelopes	At surgery	Recurrence, ROM, apprehension sign, relocation test, Constant, Rowe	NR
Syed 2010	USA	Soft tissue fluid retention after shoulder arthroscopy	Fenestrated outflow cannula	14	0	Conventional cannula	14	0	Sealed envelopes	NR	Fluid weight gain	Patients
Tan 2006	UK	Recurrent traumatic anterior instability	Arthroscopic Bankart repair with nonabsorbable anchor	65	2	Arthroscopic Bankart repair with absorbable anchor	65	4	Sealed envelopes	At surgery	OISS, pain and instability VAS, SF-12, recurrence	Patients and Assessors
Taverna 2007	Italy	Chronic supraspinatus tendinosis	Arthroscopic subacromial decompression	30	0	Radiofrequency-based plasma microtenotomy	30	0	Sealed envelopes	Just before surgery	Pain VAS, Constant, ASES, UCLA, SF-36	Patients and Assessor (physician)
Wintzell 1996	Sweden	Acute traumatic primary anterior dislocation	Arthroscopic lavage	15	0	Conservative treatment	15	0	NR	NR	Recurrenc, apprehension test, ROM, Lysholm score	Assessor

*The references are listed in Additional file 2.

^†^Dropouts/withdrawals were mentioned but the exact number in each group was not clear in the article.

*Abbreviations* in alphabetical order:

*ADL* Activities of Daily Living, *ASES* American Shoulder and Elbow Surgeons shoulder score, *ASOSS* Athletic Shoulder Outcome Scoring System, *ATH* Athletic Shoulder Scoring System score, *Constant* Constant shoulder score, *CRP* C-Reactive Protein, *CTA* Computed Tomography Arthrography, *DASH* Disabilities of the Arm, Shoulder and Hand score, *D/W* dropouts/withdrawals, *FSET* Functional Shoulder Elevation Test, *LOHS* length of hospital stay, *MRI* Magnetic Resonance Imaging, *N* number of patients randomized, *NR* not reported, *OISS* Oxford Instability Shoulder Score, *PROM* Passive Range Of Motion, *RC-QOL* Rotator Cuff Quality Of Life score, *ROM* Range Of Motion, *Rowe* Rowe shoulder score, *RTA* return to activities, *RTW* return to work, *SANE* Single Assessment Numeric Evaluation score, *SF-12* Short Form 12 survey, *SF-36* Short Form 36 survey, *SRQ* Shoulder Rating Questionnaire, *SST* Simple Shoulder Test, *UCLA* University of California-Los Angeles shoulder rating scale, *VAS* Visual Analogue Scale, *WORC* Western Ontario Rotator Cuff index, *WOSI* Western Ontario Shoulder Instability index.

### Shoulder arthroscopy

Of 130 possible RCTs obtained in the search, 80 were excluded: 24 were not intervention RCTs (matched cohort or cross-sectional studies, non-clinical RCTs, RCT protocols), 10 were systematic reviews or meta-analyses, 32 involved anesthesia or postoperative analgesia, 7 involved physiotherapy/postoperative rehabilitation, 6 were subsequent publications of same RCT, and 1 was not intervention through arthroscopy (after review of full-text and contact with the author). Thus, 50 shoulder intervention RCTs were included (Figure 1; Additional file 2). The 50 RCTs (Table 1) involved rotator cuff tears (n = 22), instability (n = 14), impingement (n = 9), and other conditions (n = 5). The interventions compared were different arthroscopic procedures (n = 31), arthroscopic versus open procedures (n = 12), and arthroscopic procedure versus nonoperative treatment (n = 7). The median number of participants was 60 (range 17–150).

### Trial quality

Of the 4 wrist studies 2 used inappropriate randomization methods and the remaining 2 stated use of “sealed envelopes” but without reporting how the randomization sequence was generated. None of the studies reported blinding and only 2 provided information about dropouts/withdrawals. In the 50 shoulder RCTs, the randomization method was described and appropriate in 25 (50%), described but inappropriate in 18 (36%) and was not described in 7 (14%). Blinding using an appropriate method was reported in 23 studies (46%), blinding was reported but the method was inappropriate in 5 (10%) and blinding was not reported in 22 studies (44%). Dropouts/withdrawals were described in 41 (82%).

The median modified Jadad score for the wrist arthroscopy intervention RCTs was 0.5 (range 0–1) and for the shoulder arthroscopy intervention RCTs was 3.0 (range 0–5). The quality of the shoulder RCTs was significantly higher than that for the wrist RCTs (p = 0.012).

### Discussion

Our study shows that despite the increasing use of wrist arthroscopy in the treatment of various wrist disorders the efficacy of arthroscopically performed interventions has only been studied in 4 quasi-randomized studies. This can be compared to 50 randomized or quasi-randomized studies of significantly higher quality for arthroscopically performed shoulder interventions, yet both procedures were first described in the literature in the late 1970s.

Since their introduction as diagnostic tools, both wrist and shoulder arthroscopy have undergone technical advancement and broader clinical applications. However, they appear to diverge in the extent to which they have been evaluated scientifically. It might be argued that shoulder disorders are more common and therefore it would be easier to conduct randomized trials. However, wrist arthroscopy is being used for several wrist disorders that are relatively common. Besides, multicenter trials can be conducted when a condition is not that common to allow enrollment of an adequate number of patients in a reasonable time. In contrast to wrist arthroscopy, endoscopic carpal tunnel release, an arthroscopic procedure, first described in the literature in the late 1980s, has been evaluated in numerous intervention RCTs, including a number of high quality trials as judged by the Cochrane reviews [10]. Also, our review of shoulder arthroscopy RCTs shows that it is possible to conduct good-quality surgical intervention trials involving arthroscopy.

Arthroscopic interventions are now used for new areas in upper extremity surgery such as thumb carpometacarpal osteoarthritis, a common condition, still without evidence from randomized studies. Because conducting good-quality surgical RCTs, with the many factors involved, is generally more difficult than pharmaceutical trials, proposals have been presented recently to facilitate surgical trials [11,12]. The lack of high-level evidence, based on good-quality randomized trials, to support the large number of surgical interventions performed through wrist arthroscopy should be a concern not only to health care payers and providers but also to patients.

Like other quality assessment systems, the Jadad scale has its limitations. Although the scale considers the appropriateness of the randomization method, which is fundamental, it does not include concealment. We have however extracted the data concerning concealment for each trial, when such data were reported (Table 1). Further, blinding of patients may not be feasible in surgical interventions. However, we also considered blinding of outcome assessors and this should be feasible in surgical trials. Another limitation is the possible existence of RCTs that the search did not capture. However, we do not believe that the search missed any eligible wrist intervention RCTs.

It is highly unlikely that a study that had used blinding or achieved complete follow-up with no drop-outs or withdrawals would not report these in the published article as important strengths. We considered studies that only mentioned using “sealed envelopes” without specifying how the randomization sequence was generated (2 wrist studies and 11 shoulder studies) as not having reported the randomization method and thus were not awarded any points for randomization. Even if we assume that these studies had used appropriate methods in generating the randomization sequence the results would be similar (median score 1.5 vs 3.0; p = 0.041).

In our search we could not find any previous studies that have assessed the quality of intervention trials involving wrist arthroscopy. With regard to RCTs that involved shoulder arthroscopy, there have been systematic reviews of intervention trials for specific shoulder disorders that included interventions done through arthroscopy. Most of these reviews used different quality scales and therefore could not be compared directly with our study. For example, a systematic review of interventions for anterior shoulder instability assessed the quality of 3 trials with a 12-item scale that included concealment and blinding (each item scored 0, 1 or 2 for a best possible total score of 24 points) giving them a score of 17, 16 and 15, respectively [13]. The modified Jadad score for the same 3 trials in our study was 3, 2 and 0, respectively, which reflects the fact that the modified Jadad scale focuses on the unambiguous reporting of the fundamental issues of randomization, blinding and drop-outs/withdrawals.

In one previous systematic review that used the original Jadad scale in assessing the quality of 54 rotator cuff RCTs published from 2001 to 2011, the mean Jadad score was 3.0 [14]. The authors concluded that most trials were of high quality (66% had a Jadad score >3.0) but because almost two-thirds of the high-quality studies were nonoperative trials they suggested that the rotator cuff literature lacks high quality RCTs that are relevant to surgical clinical practice [14]. In another report based on the “comparative effectiveness of nonoperative and operative treatments for rotator cuff tears” systematic review of literature from 1990 to 2009, the authors concluded that the “RCT literature was of particularly low quality with high risk of bias from the manner in which the studies had been conducted” [15]. Thus, despite our finding that most intervention RCTs involving shoulder arthroscopy were of significantly higher quality than the very few wrist arthroscopy trials that have been performed, there is need for further improved shoulder surgical RCTs. For example, six RCTs (published since 2002) that have assessed the efficacy of knee arthroscopy in the treatment of osteoarthritis [16] are probably of substantially higher quality than most shoulder arthroscopy RCTs.

In a study that estimated the number of upper extremity ambulatory procedures performed in the United States in 2006, including wrist and shoulder arthroscopic interventions, the authors concluded that the resources utilized by these procedures are substantial and suggested that evidence-based clinical indications and outcomes of many of these upper extremity procedures remain poorly defined [3]. For interventions involving wrist arthroscopy, our systematic review shows that there is currently a lack of good evidence supporting the efficacy of these procedures.

## Conclusions

This systematic review revealed that the efficacy of arthroscopically performed wrist interventions has been studied in only 4 quasi-randomized studies compared to 50 randomized or quasi-randomized studies of significantly higher quality assessing interventions performed through shoulder arthroscopy. In order to advance evidence-based care of patients with wrist disorders, there is a need for high-quality RCTs designed to assess the efficacy of the procedures currently performed through wrist arthroscopy.

## Competing interests

The authors of this manuscript declare that they have no financial or non-financial competing interests.

## Authors’ contributions

IA and RSR designed the study. RSR performed the search. KT, RSR and IA performed the review. KT and IA performed the statistical analysis. KT and IA prepared the initial draft of the manuscript. All authors read and approved the final version.

## Pre-publication history

The pre-publication history for this paper can be accessed here:

http://www.biomedcentral.com/1471-2474/15/252/prepub

## Supplementary Material

Additional file 1 Search details for randomized controlled trials (RCTs) involving wrist arthroscopy or shoulder arthroscopy. Click here for file

Additional file 2 The randomized controlled trials in which at least one arm involved intervention performed through wrist arthroscopy or shoulder arthroscopy, included in the systematic review. Click here for file
