# Supplementary material for: Intervention randomized controlled trials involving wrist and shoulder arthroscopy: a systematic review
Source: BMC Musculoskelet Disord. 2014 Jul 25;15:252. doi: 10.1186/1471-2474-15-252 (PMC4123827; doi:10.1186/1471-2474-15-252)
Supplement: Additional file 1 — Search details for randomized controlled trials (RCTs) involving wrist arthroscopy or shoulder arthroscopy. [file 1471-2474-15-252-S1.doc]

**Additional file 1.** Search details for randomized controlled trials (RCTs) involving wrist arthroscopy or shoulder arthroscopy

**Wrist arthroscopy RCTs:**

((“wrist"[MeSH Terms] OR "wrist"[All Fields] OR "wrist joint"[MeSH Terms] OR ("wrist"[All Fields] AND "joint"[All Fields]) OR "wrist joint"[All Fields]) AND ("arthroscopy"[MeSH Terms] OR "arthroscopy"[All Fields])) AND (("random allocation"[MeSH Terms] OR ("random"[All Fields] AND "allocation"[All Fields]) OR "random allocation"[All Fields] OR "randomized"[All Fields]) AND ("clinical trials as topic"[MeSH Terms] OR ("clinical"[All Fields] AND "trials"[All Fields] AND "topic"[All Fields]) OR "clinical trials as topic"[All Fields] OR "trial"[All Fields]).

**Shoulder arthroscopy RCTs:**

(("shoulder"[MeSH Terms] OR "shoulder"[All Fields]) AND ("arthroscopy"[MeSH Terms] OR "arthroscopy"[All Fields])) AND (("random allocation"[MeSH Terms] OR ("random"[All Fields] AND "allocation"[All Fields]) OR "random allocation"[All Fields] OR "randomized"[All Fields]) AND ("clinical trials as topic"[MeSH Terms] OR ("clinical"[All Fields] AND "trials"[All Fields] AND "topic"[All Fields]) OR "clinical trials as topic"[All Fields] OR "trial"[All Fields])).
