# Supplementary material for: Intervention randomized controlled trials involving wrist and shoulder arthroscopy: a systematic review
Source: BMC Musculoskelet Disord. 2014 Jul 25;15:252. doi: 10.1186/1471-2474-15-252 (PMC4123827; doi:10.1186/1471-2474-15-252)
Supplement: Additional file 2 — The randomized controlled trials in which at least one arm involved intervention performed through wrist arthroscopy or shoulder arthroscopy, included in the systematic review. [file 1471-2474-15-252-S2.pdf]

Additional file 2 The randomized controlled trials in which at least one arm involved intervention performed through wrist arthroscopy or shoulder arthroscopy, included in the systematic review (the 4 wrist and 50 shoulder references are listed in alphabetical order of first author).

Wrist

1. Leblebicioglu G, Doral MN, Atay A, Tetik O, Whipple TL: **Open treatment of stage III Kienböck's disease with lunate revascularization compared with arthroscopic treatment without revascularization**. *Arthroscopy* 2003, **19:**117–130.

2. Kang L, Akelman E, Weiss AP: **Arthroscopic versus open dorsal ganglion excision: a prospective, randomized comparison of rates of recurrence and of residual pain**. *J Hand Surg Am* 2008, **33:**471–475.

3. Rocchi L, Canal A, Fanfani F, Catalano F: **Articular ganglia of the volar aspect of the wrist: arthroscopic resection compared with open excision. A prospective randomised study**. *Scand J Plast Reconstr Surg Hand Surg* 2008, **42:**253–259.

4. Varitimidis SE, Basdekis GK, Dailiana ZH, Hantes ME, Bargiotas K, Malizos K: **Treatment of intra-articular fractures of the distal radius: fluoroscopic or arthroscopic reduction?** *J Bone Joint Surg Br* 2008, **90:**778–785.

Shoulder

5. Archetti NN, Tamaoki MJ, Lenza M, dos Santos JB, Matsumoto MH, Faloppa F, Belloti JC: **Treatment of Bankart lesions in traumatic anterior instability of the shoulder: a randomized controlled trial comparing arthroscopy and open techniques.** *Arthroscopy* 2012, **28:**900–908.

6. Barber FA, Burns JP, Deutsch A, Labbe MR, Litchfield RB: **A prospective, randomized evaluation of acellular human dermal matrix augmentation for arthroscopic rotator cuff repair**. *Arthroscopy* 2012, **28:**8–15.

7. Berth A, Neumann W, Awiszus F, Pap G: **Massive rotator cuff tears: functional outcome after debridement or arthroscopic partial repair.** *J Orthop Traumatol* 2010, **11:**13–20.

8. Bottoni CR, Wilckens JH, DeBerardino TM, D'Alleyrand JC, Rooney RC, Harpstrite JK, Arciero RA: **A prospective, randomized evaluation of arthroscopic stabilization versus nonoperative treatment in patients with acute, traumatic, first-time shoulder dislocations.** *Am J Sports Med* 2002, **30:**576–580.

9. Bottoni CR, Smith EL, Berkowitz MJ, Towle RB, Moore JH: **Arthroscopic versus open shoulder stabilization for recurrent anterior instability: a prospective randomized clinical trial.** *Am J Sports Med* 2006, **34:**1730–1737.

10. Brox JI, Staff PH, Ljunggren AE, Brevik JI: **Arthroscopic surgery compared with supervised exercises in patients with rotator cuff disease (stage II impingement syndrome).** *BMJ* 1993, **307:**899–903.

11. Burks RT, Crim J, Brown N, Fink B, Greis PE: **A prospective randomized clinical trial comparing arthroscopic single- and double-row rotator cuff repair: magnetic resonance imaging and early clinical evaluation.** *Am J Sports Med* 2009, **37:**674–682.

12. Charron KM, Schepsis AA, Voloshin I: **Arthroscopic distal clavicle resection in athletes: a prospective comparison of the direct and indirect approach.** *Am J Sports Med* 2007, **35:**53–58.

13. Chen J, Chen S, Li Y, Hua Y, Li H: **Is the extended release of the inferior glenohumeral ligament necessary for frozen shoulder?** *Arthroscopy* 2010, **26:**529–535.

14. De Carli A, Vadalà A, Perugia D, Frate L, Iorio C, Fabbri M, Ferretti A: **Shoulder adhesive capsulitis: manipulation and arthroscopic arthrolysis or intra-articular steroid injections?** *Int Orthop* 2012, **36:**101–106.

15. Dezaly C, Sirveaux F, Philippe R, Wein-Remy F, Sedaghatian J, Roche O, Molé D: **Arthroscopic treatment of rotator cuff tear in the over-60s: repair is preferable to isolated acromioplasty-tenotomy in the short term.** *Orthop Traumatol Surg Res* 2011, **97:**S125–S130.

16. Elmlund AO, Kartus J, Rostgard-Christensen L, Sernert N, Magnusson L, Ejerhed L: **A 7-year prospective, randomized, clinical, and radiographic study after arthroscopic Bankart reconstruction using 2 different types of absorbable tack.** *Am J Sports Med* 2009, **37:**1930–1937.

17. Fabbriciani C, Milano G, Demontis A, Fadda S, Ziranu F, Mulas PD: **Arthroscopic versus open treatment of Bankart lesion of the shoulder: a prospective randomized study.** *Arthroscopy* 2004, **20:**456–462.

18. Franceschi F, Longo UG, Ruzzini L, Rizzello G, Maffulli N, Denaro V: **No advantages in repairing a type II superior labrum anterior and posterior (SLAP) lesion when associated with rotator cuff repair in patients over age 50: a randomized controlled trial.** *Am J Sports Med* 2008, **36:**247–253.

19. Freedman BA, Javernick MA, O'Brien FP, Ross AE, Doukas WC: **Arthroscopic versus open distal clavicle excision: comparative results at six months and one year from a randomized, prospective clinical trial.** *J Shoulder Elbow Surg* 2007, **16:**413–418.

20. Gartsman GM, O'connor DP: **Arthroscopic rotator cuff repair with and without arthroscopic subacromial decompression: a prospective, randomized study of one-year outcomes.** *J Shoulder Elbow Surg* 2004, **13:**424–426.

21. Grasso A, Milano G, Salvatore M, Falcone G, Deriu L, Fabbriciani C: **Single-row versus double-row arthroscopic rotator cuff repair: a prospective randomized clinical study.** *Arthroscopy* 2009, **25:**4–12.

22. Gumina S, Campagna V, Ferrazza G, Giannicola G, Fratalocchi F, Milani A, Postacchini F: **Use of platelet-leukocyte membrane in arthroscopic repair of large rotator cuff tears: a prospective randomized study.** *J Bone Joint Surg Am* 2012, **94:**1345–1352.

23. Haahr JP, Østergaard S, Dalsgaard J, Norup K, Frost P, Lausen S, Holm EA, Andersen JH: **Exercises versus arthroscopic decompression in patients with subacromial impingement: a randomised, controlled study in 90 cases with a one year follow up.** *Ann Rheum Dis* 2005, **64:**760–764.

24. Henkus HE, de Witte PB, Nelissen RG, Brand R, van Arkel ER: **Bursectomy compared with acromioplasty in the management of subacromial impingement syndrome: a prospective randomised study.** *J Bone Joint Surg Br* 2009, **91:**504–510.

25. Hiemstra LA, Sasyniuk TM, Mohtadi NG, Fick GH: **Shoulder strength after open versus arthroscopic stabilization.** *Am J Sports Med* 2008, **36:**861–867.

26. Husby T, Haugstvedt JR, Brandt M, Holm I, Steen H: **Open versus arthroscopic subacromial decompression: a prospective, randomized study of 34 patients followed for 8 years.** *Acta Orthop Scand* 2003, **74:**408–414.

27. Kasten P, Keil C, Grieser T, Raiss P, Streich N, Loew M: **Prospective randomised comparison of arthroscopic versus mini-open rotator cuff repair of the supraspinatus tendon.** *Int Orthop* 2011, **35:**1663–1670.

28. Ketola S, Lehtinen J, Arnala I, Nissinen M, Westenius H, Sintonen H, Aronen P, Konttinen YT, Malmivaara A, Rousi T: **Does arthroscopic acromioplasty provide any additional value in the treatment of shoulder impingement syndrome?: a two-year randomised controlled trial.** *J Bone Joint Surg Br* 2009, **91:**1326–1334.

29. Kim J, Chung J, Ok H: **Asymptomatic acromioclavicular joint arthritis in arthroscopic rotator cuff tendon repair: a prospective randomized comparison study.** *Arch Orthop Trauma Surg* 2011, **131:**363–369.

30. Kirkley A, Griffin S, Richards C, Miniaci A, Mohtadi N: **Prospective randomized clinical trial comparing the effectiveness of immediate arthroscopic stabilization versus immobilization and rehabilitation in first traumatic anterior dislocations of the shoulder.** *Arthroscopy* 1999, **15:**507–514.

31. Koh KH, Kang KC, Lim TK, Shon MS, Yoo JC: **Prospective randomized clinical trial of single- versus double-row suture anchor repair in 2- to 4-cm rotator cuff tears: clinical and magnetic resonance imaging results.** *Arthroscopy* 2011, 27:453–462.

32. Lindh M, Norlin R: **Arthroscopic subacromial decompression versus open acromioplasty: a two-year follow-up study.** *Clin Orthop Relat Res* 1993, **290:**174–176.

33. Ma HL, Chiang ER, Wu HT, Hung SC, Wang ST, Liu CL, Chen TH: **Clinical outcome and imaging of arthroscopic single-row and double-row rotator cuff repair: a prospective randomized trial.** *Arthroscopy* 2012, **28:**16–24.

34. MacDonald P, McRae S, Leiter J, Mascarenhas R, Lapner P: **Arthroscopic rotator cuff repair with and without acromioplasty in the treatment of full-thickness rotator cuff tears: a multicenter, randomized controlled trial.** *J Bone Joint Surg Am* 2011, **93:**1953–1960.

35. Magnusson L, Ejerhed L, Rostgård-Christensen L, Sernert N, Eriksson R, Karlsson J, Kartus JT: **A prospective, randomized, clinical and radiographic study after arthroscopic Bankart reconstruction using 2 different types of absorbable tacks.** *Arthroscopy* 2006, **22:**143–151.

36. Milano G, Grasso A, Salvatore M, Zarelli D, Deriu L, Fabbriciani C: **Arthroscopic rotator cuff repair with and without subacromial decompression: a prospective randomized study.** *Arthroscopy* 2007, **23:**81–88.

37. Milano G, Grasso A, Salvatore M, Saccomanno MF, Deriu L, Fabbriciani C: **Arthroscopic rotator cuff repair with metal and biodegradable suture anchors: a prospective randomized study.** *Arthroscopy* 2010, **26:**S112–S119.

38. Milano G, Grasso A, Santagada DA, Saccomanno MF, Deriu L, Fabbriciani C: **Comparison between metal and biodegradable suture anchors in the arthroscopic treatment of traumatic anterior shoulder instability: a prospective randomized study.** *Knee Surg Sports Traumatol Arthrosc* 2010, **18:**1785–1791.

39. Mohtadi NG, Hollinshead RM, Sasyniuk TM, Fletcher JA, Chan DS, Li FX: **A randomized clinical trial comparing open to arthroscopic acromioplasty with mini-open rotator cuff repair for full-thickness rotator cuff tears: disease-specific quality of life outcome at an average 2-year follow-up.** *Am J Sports Med* 2008, **36:**1043–1051.

40. Monteiro GC, Ejnisman B, Andreoli CV, de Castro PA, Cohen M: **Absorbable versus nonabsorbable sutures for the arthroscopic treatment of anterior shoulder instability in athletes: a prospective randomized study.** *Arthroscopy* 2008, **24:**697–703.

41. Oh CH, Oh JH, Kim SH, Cho JH, Yoon JP, Kim JY: **Effectiveness of subacromial anti-adhesive agent injection after arthroscopic rotator cuff repair: prospective randomized comparison study.** *Clin Orthop Surg* 2011, **3:**55–61.

42. Randelli P, Arrigoni P, Ragone V, Aliprandi A, Cabitza P: **Platelet rich plasma in arthroscopic rotator cuff repair: a prospective RCT study, 2-year follow-up.** *J Shoulder Elbow Surg* 2011, **20:**518–528.

43. Robinson CM, Jenkins PJ, White TO, Ker A, Will E: **Primary arthroscopic stabilization for a first-time anterior dislocation of the shoulder. A randomized, double-blind trial.** *J Bone Joint Surg Am* 2008, **90:**708–721.

44. Rodeo SA, Delos D, Williams RJ, Adler RS, Pearle A, Warren RF: **The effect of platelet-rich fibrin matrix on rotator cuff tendon healing: a prospective, randomized clinical study.** *Am J Sports Med* 2012, **40:**1234–1241.

45. Sachs RA, Stone ML, Devine S: **Open vs. arthroscopic acromioplasty: a prospective, randomized study.** *Arthroscopy* 1994, **10:**248–254.

46. Shin SJ, Oh JH, Chung SW, Song MH: **The efficacy of acromioplasty in the arthroscopic repair of small- to medium-sized rotator cuff tears without acromial spur: prospective comparative study.** *Arthroscopy* 2012, **28:**628–635.

47. Shin SJ: **A comparison of 2 repair techniques for partial-thickness articular-sided rotator cuff tears.** *Arthroscopy* 2012, **28:**25–33.

48. Silberberg JM, Moya-Angeler J, Martin E, Leyes M, Forriol F: **Vertical versus horizontal suture configuration for the repair of isolated type II SLAP lesion through a single anterior portal: a randomized controlled trial.** *Arthroscopy* 2011, **27:**1605–1613.

49. Spangehl MJ, Hawkins RH, McCormack RG, Loomer RL: **Arthroscopic versus open acromioplasty: a prospective, randomized, blinded study.** *J Shoulder Elbow Surg* 2002, 11:101–107.

50. Sperber A, Hamberg P, Karlsson J, Sward L, Wredmark T: **Comparison of an arthroscopic and an open procedure for posttraumatic instability of the shoulder: a prospective, randomized multicenter study.** *J Shoulder Elbow Surg* 2001, **10:**105–108.

51. Syed HM, Gillham SB, Jobe CM, Phipatanakul WP, Wongworawat MD: **Fenestrated cannulae with outflow reduces fluid gain in shoulder arthroscopy.** *Clin Orthop Relat Res* 2010, **468:**158–162.

52. Tan CK, Guisasola I, Machani B, Kemp G, Sinopidis C, Brownson P, Frostick S: **Arthroscopic stabilization of the shoulder: a prospective randomized study of absorbable versus nonabsorbable suture anchors.** *Arthroscopy* 2006, **22:**716–720.

53. Taverna E, Battistella F, Sansone V, Perfetti C, Tasto JP: **Radiofrequency-based plasma microtenotomy compared with arthroscopic subacromial decompression yields equivalent outcomes for rotator cuff tendinosis.** *Arthroscopy* 2007, **23:**1042–1051.

54. Wintzell G, Haglund-Akerlind Y, Tidermark J, Wredmark T, Eriksson E: **A prospective controlled randomized study of arthroscopic lavage in acute primary anterior dislocation of the shoulder: one-year follow-up.** *Knee Surg Sports Traumatol Arthrosc* 1996, **4:**43–47.
